# Single Centre Experience with Minimally Invasive Aortic Valve Replacement versus Conventional Full Sternotomy Approach - A Propensity Match Analysis

**DOI:** 10.1186/1749-8090-10-S1-A109

**Published:** 2015-12-16

**Authors:** Karim Morcos, Cathy Johnman, Cristiano Spadaccio, Sadia Aftab, Fraser Sutherland

**Affiliations:** 1 Department of Cardiothoracic Surgery, Golden Jubilee National Hospital, Glasgow, Scotland, UK; 2 Institute of Health and Wellbeing, College of Medical, Veterinary and Life Sciences, Public Health University of Glasgow, Glasgow, Scotland, UK

## Background/Introduction

Minimally invasive approach to aortic valve replacement (AVR) is increasingly accepted as a valid alternative to conventional full sternotomy (FS-AVR), as reduces operative trauma with the final aim to improve the postoperative outcomes.

## Aims/Objectives

The aim of our study is to compare short term clinical outcomes after minimally invasive AVR (mini-AVR) with outcomes following FS-AVR in the same institution.

## Method

Between December 2010 and March 2012 627 patients underwent isolated AVR were retrospectively included in two groups: 599 patients underwent FS-AVR sternotomy (Group A), while 28 underwent minimally invasive procedure (Group B). Mini-AVR was performed through a 6 cm upper midline incision with reverse 'J' manubriotomy carried into the right third intercostal space. Venous drainage for cardiopulmonary bypass was achieved alternatively percutaneously or with a flat two stage venous cannula with vacuum assist. Primary endpoint was peri-procedural mortality; secondary endpoints were overall postoperative complications, major adverse cardiac-related complication, use of blood products and need for transfusions, bypass time and cross-clamp time, ventilation time and length of stay in hospital. Propensity score match analysis was performed to avoid selection biases and equalize confounding preoperative variables.

## Results

After propensity score match, no statistical significant difference was found in peri-procedural mortality rate (p > 0.05), mean bypass and cross clamp times. Minimally invasive AVR was associated with a significant reduced need for transfusion (p = 0.003), as well as postoperative cardiac and non-cardiac complications. A trend towards lower mean ventilation times, ICU stay and hospital stay in the mini-AVR group was also detected, but failed to reach statistical significance.

## Discussion/Conclusion

Initial results with minimally invasive AVR are associated with significantly reduced blood loss, reduced blood transfusion and a trend towards less ventilation time, ICU stay and hospital stay. Postoperative cosmetic results were much better in the minimally invasive group.

